# Proteomic Analysis of *Sporothrix schenckii* Exposed to Oxidative Stress Induced by Hydrogen Peroxide

**DOI:** 10.3390/pathogens11020230

**Published:** 2022-02-10

**Authors:** Dulce O. Saucedo-Campa, Ana L. Martínez-Rocha, Emmanuel Ríos-Castro, Carlos A. Alba-Fierro, Miguel A. Escobedo-Bretado, Mayra Cuéllar-Cruz, Estela Ruiz-Baca

**Affiliations:** 1 Facultad de Ciencias Químicas (Unidad Durango), Universidad Juárez del Estado de Durango, Av. Veterinaria S/N, Durango 34120, Mexico; qfb.saucam@gmail.com (D.O.S.-C.); analilia.martinez@ujed.mx (A.L.M.-R.); carlos.alba@ujed.mx (C.A.A.-F.); miguel.escobedo@ujed.mx (M.A.E.-B.); 2 Centro de Investigación y de Estudios Avanzados del I.P.N., Unidad de Genómica, Proteómica y Metabolómica, LaNSE, Ciudad de Mexico 07360, Mexico; eriosc@cinvestav.mx; 3 Departamento de Biología, División de Ciencias Naturales y Exactas, Campus Guanajuato, Universidad de Guanajuato, Noria Alta S/N, Guanajuato 36050, Mexico; mcuellar@ugto.mx

**Keywords:** *Sporothrix schenckii*, cell wall proteins, moonlighting proteins, oxidative stress, H_2_O_2_

## Abstract

*Sporothrix schenckii* modulates the expression of its cell wall proteins (CWPs) in response to reactive oxygen species (ROS) generated by the phagocytic cells of the human host, which allows it to evade and escape the immune system. In this study, we performed a comparative proteomic analysis of the CW of *S. schenckii* after exposure and nonexposure to H_2_O_2_. Several CWPs involved in CW remodeling and fungal pathogenesis that modulated their expression in response to this oxidizing agent were identified, as were a number of antioxidant enzymes and atypical CWPs, called moonlighting proteins, such as the Hsp70-5, lipase 1 (Lip1), enolase (Eno), and pyruvate kinase (Pk). Moreover, RT-qPCR assays demonstrated that the transcription of genes *HSP70-5*, *LIP1*, *ENO*, and *PK* is regulated in response to the oxidant. The results indicated that *S. schenckii* differentially expressed CWPs to confer protection against ROS upon this fungus. Furthermore, among these proteins, antioxidant enzymes and interestingly, moonlighting-like CWPs play a role in protecting the fungus from oxidative stress (OS), allowing it to infect human host cells.

## 1. Introduction

Sporotrichosis is an emergent subcutaneous mycosis that affects both humans and animals. It is a cosmopolitan disease, reported to be endemic in the tropical and subtropical regions of Latin America, Africa and Asia [1,2,3]. *Sporothrix schenckii*, which belongs to the pathogenic clade of the genus *Sporothrix*, is the species most frequently associated with this disease [4].

The human body’s resistance to pathogenic fungi depends largely on the behavior of the phagocytic cells of the innate immune system, especially on that of neutrophils and macrophages. Phagocytes are very efficient at recognizing fungi, mainly by recognizing specific molecules of their cell surface known as pathogen-associated molecular pattern molecules (PAMPs) through pattern recognition receptors (PRRs). When phagocytes recognize fungal cells, they absorb and degrade them inside phagosomal compartments. Myriad agents contribute to the inhibitory and destructive properties of phagolysosomes, including enzymes that produce reactive oxygen species (ROS) and reactive nitrogen species (RNS) [5,6,7]. During the respiratory burst, the main oxygen or nitrogen species produced are hydrogen peroxide (H_2_O_2_), radical superoxide (O_2_^•−^), nitric oxide (NO^•^), and hypochlorous acid (HOCl). For example, macrophages can generate almost 60 μM NO^•^ and up to 14 mM H_2_O_2_ [6]. The superoxide radical O_2_^•−^ is the main ROS produced; it gives rise to other biologically important ROS, such as H_2_O_2_, the hydroxyl radical (^•^OH), the peroxyl radical and singlet or individual oxygen [8]. Nevertheless, in basal conditions, O_2_^•−^ and H_2_O_2_ make up most of the ROS produced inside the cell. Of the latter two ROS, H_2_O_2_ is considered the most important one, since it is reduced by the Fenton reaction [9] to ^•^OH in the presence of Fe^2+^, and the ^•^OH radical is extremely reactive, attacking all biomolecules [8]. Hence, both O_2_^•−^ and H_2_O_2_ are the most important ROS to be studied, because knowledge of the mechanism of action of ROS on the biomolecules of fungal cells is necessary to identify host cellular responses to ROS. The toxic environment of phagolysosomes activates a wide range of survival mechanisms in the fungal cells that mitigate the effect of oxidative and nitrosative stress damage, the pH, and other types of stress that can kill pathogenic fungi. When fungal intracellular survival strategies allow pathogens to survive within phagocytes, they eventually escape, spread, and infect distant tissue [5,6].

During the infectious process, the cell wall (CW) acts as the interface between the fungus and the host. The fungal CW is a dynamic structure which is essential for cell viability, morphogenesis, and pathogenesis. Pathogenic fungi can modify the structure of their CWs in response to signals from the host in a way that allows them to evade the host’s immune system or hyperactivate it [10]. Cell wall proteins (CWPs) play an important role in the interaction between a fungus and its host; they are involved in the fungal adhesion to different surfaces, in providing protection against harmful environmental hazards, in camouflaging the fungal cells from phagocytes, and even in remodeling the CW in response to growth, morphological changes, or adaptations to changes in the external environment [10,11]. Interestingly, numerous CWPs are moonlighting-like proteins. These proteins have a dual location, are noncovalently bound to the CW, and are also found in the cytoplasm, where they play different metabolic roles [12].

In order to remain in the host and cause damage, *S. schenckii* has to adhere to its target cell through CWPs while resisting the oxidative stress (OS) caused by ROS and RNS produced by host phagocytes. In addition to detoxification systems, pathogenic fungi have other mechanisms that allow them to survive and evade their host’s immune systems. In recent studies, we identified 13 CWPs involved in the oxidative stress response (OSR) induced by menadione (a source of O_2_^•−^) in yeast cells of *S. schenckii*; we believe that these proteins could help the fungus survive inside the host cells [13]. However, the way *S. schenckii* responds to other ROS such as H_2_O_2_ has not been evaluated. Despite the fact that H_2_O_2_ is not very reactive, it can cross biological membranes [14] and, in the presence of reduced transition metals, is a precursor of ^•^OH, one of the strongest oxidants in nature [15]. Interestingly, even though O_2_^•−^ and H_2_O_2_ are both ROS, and it would be expected that they have similar functions in cells, it has been shown that their functions differ. For example, it was found that O_2_^•−^ induces proliferation in vascular smooth muscle cells while H_2_O_2_ causes apoptosis [16]. It has also been reported that H_2_O_2_ preferentially serves as a messenger molecule through oxidative modification of signaling proteins.

Due to the significance of H_2_O_2_, it is important to identify the CWPs of *S. schenckii* that participate in the OSR to this oxidizing agent. This could make it possible to formulate an initial explanation of whether this pathogen responds differentially to H_2_O_2_ relative to O_2_^•−^. Based on the previous description, the main objective of the present study was to evaluate which CWPs of *S. schenckii* modify its expression in response to H_2_O_2_. This knowledge would help us to understand the mechanisms associated with the CW whereby this fungus can evade the host’s phagocytic cells and cause infection.

## 2. Results

### 2.1. H_2_O_2_ Susceptibility Assay

**Figure 1:**
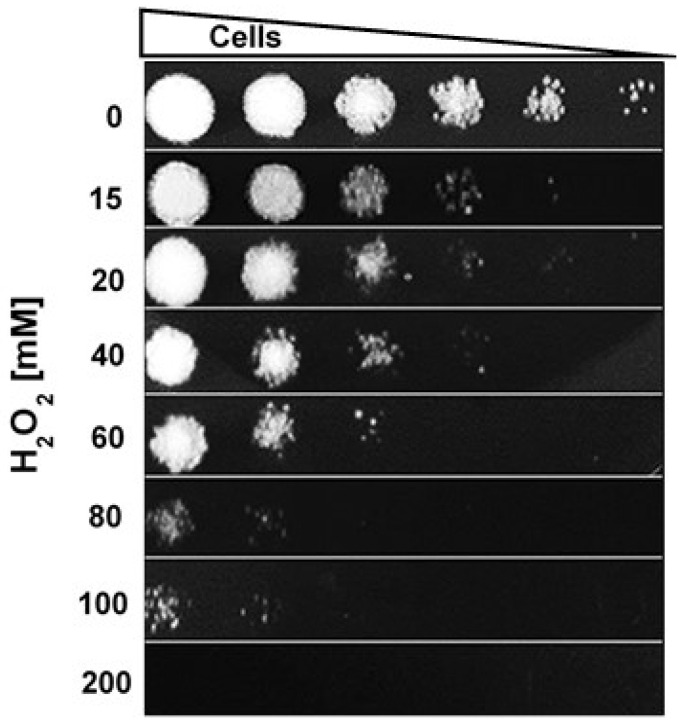
Susceptibility of *S. schenckii* to H_2_O_2_. Cultures of exponential-phase yeast cell (OD_600nm_ 0.5) were incubated under constant stirring in the presence of the indicated concentrations of H_2_O_2_ at 37 °C. Samples of these suspensions were exponentially diluted in 96-well plates and each dilution was spotted onto YPG plates that were incubated at 37 °C. Growth was inspected after 48 h.

In order to calculate the minimum lethal dose (MLD) of H_2_O_2_ for *S. schenckii*, yeast cultures of the fungus in the exponential growth phase were exposed to different concentrations (0–200 mM). Figure 1 shows that inhibition of fungal growth was be observed with a concentration of 15 mM, while 200 mM was the minimum lethal concentration.

### 2.2. Identification of CWPs in S. Schenckii

**Figure 2:**
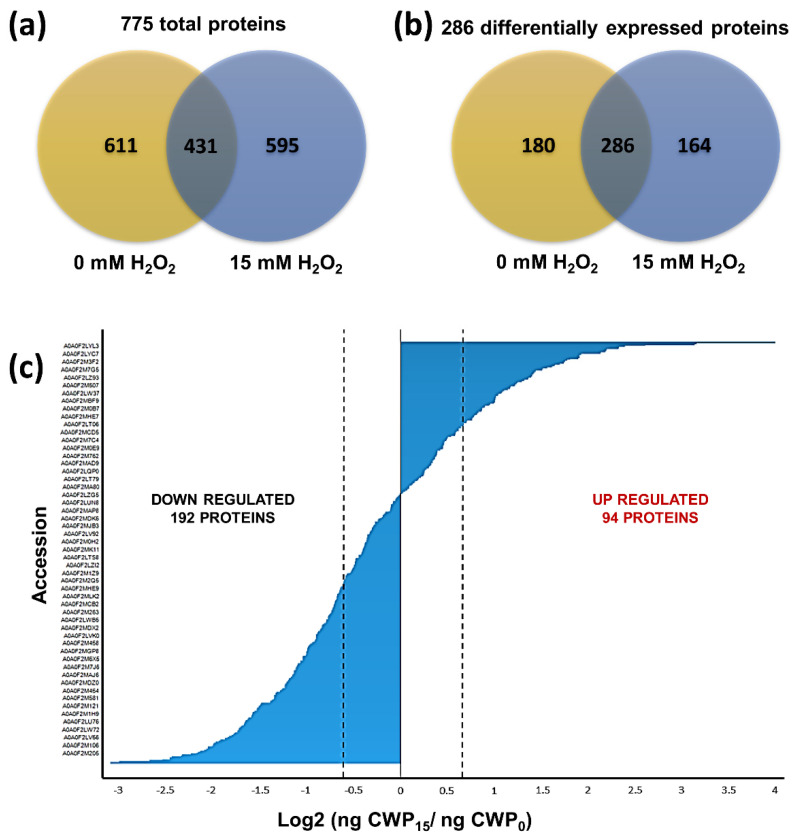
Proteins identified by LC-ESI-IMS- QTof in yeast cells of *S. schenckii* treated or not with H_2_O_2_. (**a**) Number of total proteins identified. (**b**) Number of proteins that were expressed differentially. (**c**) Number of proteins that upregulated and downregulated their expression.

Based on the results from the susceptibility assays of *S. schenckii* yeast cells to H_2_O_2_ and considering that phagocytic cells such as macrophages can generate up to 14 mM H_2_O_2_ [6], we choose to identify CWPs in cells exposed to 15 mM of the oxidizing agent (Figure 1). At this concentration, the cells are still viable, thereby ensuring that identified CWPs were involved in the response to H_2_O_2_. The CWPs were extracted using β-mercaptoethanol-SDS. The extracted proteins were analyzed using LC-ESI-IMS-QTof. A total of 15,522 peptides were identified, of which 12,575 (81.66%) had an error of ±10 ppm (Figure S1a), indicating that the spectrometer had been adequately calibrated. These peptides corresponded to 775 proteins that were identified and quantified (≥95% confidence, Protein AutoCurate Green), from which 620 had a known function (Data Set S1). The number of proteins identified in cells that were exposed and cells that were not exposed to the oxidizing agent was 595 for the former and 611 for the latter (Figure 2a). Of these proteins, 431 were present under both conditions; their dynamic range was adequate for both conditions, indicating a correct normalization during injection (Figure S1b). The main functional groups of proteins that changed their expression in response to H_2_O_2_ include proteins associated with cellular metabolism, energy production, protein synthesis, and stress response functions.

### 2.3. Differentially Expressed CWPs in S. Schenckii in the Presence of H_2_O_2_

A total of 286 proteins were differentially expressed between the exposed and nonexposed cells (Figure 2b), of which 94 were upregulated, while 192 downregulated their expression (Figure 2c). Furthermore, 164 and 180 proteins were exclusive to exposed and nonexposed cells, respectively (Data Set S1).

Twenty-eight proteins associated with the CW of *S. schenckii* that modulated their expression in response to H_2_O_2_ could be potentially involved in the OSR mechanism and confer protection to this fungus in the host’s phagocytic cells (Table 1).

Among these proteins, there are nine proteins directly related to the CW such as GPI-anchored cell wall β-1,3-endoclucanase EglC, β-glucosidase, covalently-linked cell wall protein, two β-1,3-glucanosyltransferases, glycoside hydrolase, GPI anchored cell wall protein, glycosidase crf1, and CFEM domain-containing protein which were upregulated in the presence of H_2_O_2_.

Another group of CWPs identified in this study corresponded to proteins involved in the OSR such as peroxiredoxin (Prx), two superoxide dismutases (Sod) (Cu-ZN), and thioredoxin 1 (Thx1), which were upregulated in the presence of H_2_O_2_. The third group of proteins corresponded to six glycolytic enzymes such as glyceraldehyde-3-phosphate dehydrogenase (Gapdh), enolase (Eno), pyruvate kinase (Pk), phosphoglycerate kinase (Pgk), fructose-bisphosphate aldolase (Fba), and triosephosphate isomerase (Tpi) which were downregulated in the presence of H_2_O_2_. Other metabolic enzymes such as lipase 1 (Lip1), trehalose synthase (TreS), trehalasa (Trh), alcohol dehydrogenase (Adh), citrate synthase (Cs) and α, α-trehalose-phosphate synthase (Tps1) were identified. In the presence of H_2_O_2_, Lip1, TreS, Trh, and Adh were upregulated in *S. schenckii*. The Cs and Tps1 were downregulated in the presence of H_2_O_2_. Other proteins such as Hsp70-5, elongation factor 1-β (EF-1β), and elongation factor 1-α (EF-1α) were also identified. The Hsp70-5 and elongation factor 1-β (EF-1β) were upregulated and elongation factor 1-α (EF-1α) was downregulated in the presence of H_2_O_2_.

### 2.4. Transcriptional Expression Analysis of Genes Encoding Moonlighting-Like Proteins

**Figure 3:**
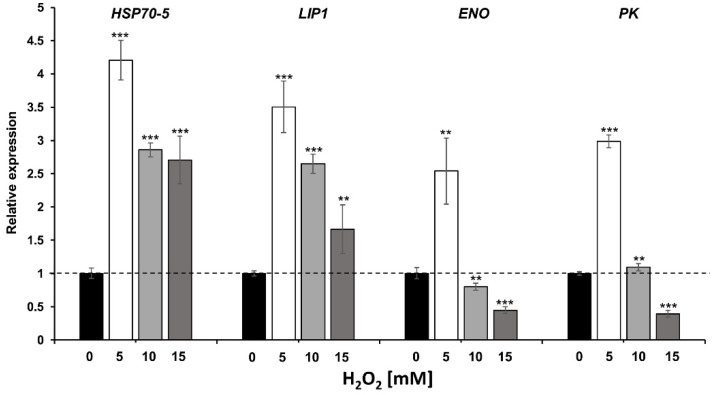
Expression analysis of *HSP70-5*, *LIP1*, *ENO*, and *PK* genes by RT-qPCR. The relative expression of the four genes selected display and upregulation of *HSP70-5* and *LIP1* genes under all conditions, while *ENO* and *PK* genes have a downregulation at 15 mM H_2_O_2_ condition. The expression was determined by the comparative method 2^−ΔΔCt^. Relative expression levels were determined using the PL6 gene as housekeeping and the 0 mM of H_2_O_2_ condition as calibrator sample. Error bars indicate the deviation calculated from three biological repetitions and the triplicate experimental data. The statistical analysis was calculated with respect to the 0 mM H_2_O_2_, using one way Anova and the Bonferroni-Holm post hoc test (significance: *** *p* < 0.001, ** *p* < 0.01, * *p* < 0.05).

Genes encoding for proteins with a differential expression involved in the interaction with the host such as Hsp70-5, Lip1, Eno, and Pk, were selected to determine the transcriptional gene expression levels by RT-qPCR. Expression of the selected genes was determined at 0, 5, 10, and 15 mM of H_2_O_2_, and assays were performed to determine the effect of H_2_O_2_ on growth at these concentrations (Figure S2). Transcriptional expression results displayed an increase in the expression of the four genes studied at the 5 mM H_2_O_2_ condition. At the 10 mM H_2_O_2_ condition an increase in the expression of *HSP70-5* and *LIP1* genes was determined, while *ENO* had a decrease in expression and *PK* did not present significant changes. The expression results at the 15 mM H_2_O_2_ condition displayed an increase in the expression of *HSP70-5* and *LIP1* genes, whereas there was a decrease in the expression of *ENO* and *PK* genes (Figure 3).

## 3. Discussion

The fungal cell surface responds to different environments, including various microenvironments within the infected host. The present study aimed to obtain this information by exposing yeast cells of *S. schenckii* in exponential phase to different concentrations of H_2_O_2_, in order to emulate the conditions faced by the fungus during the respiratory burst. This oxidizing agent began to affect the growth of the fungus at a concentration of 15 mM; the fungus was able to survive exposure to H_2_O_2_ up to 60 mM (Figure 1). There was a severe loss of viability at concentrations of 80−100 mM and a complete loss at 200 mM (Figure 1). It has been previously reported that stationary phase cells of *S. schenckii* were able to survive exposure to H_2_O_2_ up to 600 mM [17]. These data are in agreement with previous findings that stationary phase cells are more resistant to oxidants than exponential phase cells in *Candida* species [18]. Tolerance to OS in vitro in *S. schenckii* has been associated with the presence of antioxidant enzymatic mechanisms as well as other mechanisms independent of these [13,17,19].

The composition and structure of the CW of *S. schenckii* during respiratory burst is what determines whether the fungus survives or not; thus, the CW of this fungus plays a key role in the infection process. In order to identify the CWPs of *S. schenckii* that are differentially expressed in the OSR induced by H_2_O_2_, CWPs were extracted from *S. schenckii* cells that were exposed and cells that were not exposed to this oxidizing agent. The extracted proteins were then identified by LC-ESI-IMS- QTof (Supplementary Materials Data Set S1). In cells exposed to H_2_O_2_, it was possible to identify 28 proteins associated with the CW that showed differential expressions, and which have been previously reported in other studies [13,20,21,22]; these proteins might be involved in OSR mechanisms and confer protection to *S. schenckii* from OS (Table 1).

Among the differentially expressed proteins, nine are directly related to the CW in *S. schenckii* under OS induced by H_2_O_2_. The remodeling of the CW of *S. schenckii* in the OSR is very relevant, given that the activity of seven of the nine directly related CWPs identified in the present study, the GPI-anchored cell wall β-1,3-endoglucanase EglC, GPI anchored cell wall protein, two β-1,3-glucanosyltransferases, β-glucosidase, glycosidase crf1, and glycosyl hydrolase, has been associated with CW remodeling [23]. GPI-anchored cell wall β-1,3-endoglucanase EglC and glycosidase crf1, which were upregulated play an important role in the regulation of β-glucans, and a decrease in their expression has been associated with loss of virulence in phytopathogenic fungi such as *Magnaporthe oryzae* [24]. Other CWPs identified such as CFEM domain-containing protein and covalently-linked CWP were upregulated and exclusive respectively in the cells of *S. schenckii* that were exposed to the oxidant agent. Studies in *C. albicans* have demonstrated that CFEM domain protein-encoding genes are pleiotropic, influencing cell surface characteristics and biofilm formation [25]. The covalently-linked CWP has been associated to adhesion and production of biofilms in *S. cerevisiaea* and *C. glabrata* [13]. Proteins such as GPI-anchored cell wall β-1,3-endoglucanase EglC, glycosidase crf1, covalently-linked CWP, and CFEM domain-containing protein have been previously reported in *S. schenckii* under OS induced by menadione [13].

Other CWPs that were upregulated in the present work were two Sod (Cu-Zn), Trx, and Prx. The Sod (Cu-Zn) serves as the cell’s first line of defense against OS, catalyzing the conversion of superoxide ions into H_2_O_2_ and O_2_. *S. schenckii* possesses five SODs, and the role of these isoforms and their regulation during OS have not been studied [26]. Another typical OSR enzyme is Trx. *S. schenkii* possesses four Trxs and a Trx reductase (Trr) [26]. As mentioned above, the participation of Sods and Trx1 in the OSR has also been reported in *S. schenckii* in the presence of superoxide ions [13]. This is interesting because it suggests that these enzymes could be one of the main mechanisms used by *S. schenckii* to detoxify different ROS. Prx is another CWP that has been identified in *S. schenckii* in other studies on the OSR [17]. Prxs contain an active site cysteine that is sensitive to oxidation by H_2_O_2_, and this is in accord with the result obtained here that under this oxidizing agent Prx is upregulated. The upregulation of this protein in *S. schenckii* is probably explained by the fact that it represents one of the antioxidant mechanisms that helps this fungus to neutralize ROS during the respiratory burst. The expression of Prx in the CW of *S. schenckii* in the OSR is in line with other studies where it was shown that Prx is upregulated in the CW of *C. glabrata* when exposed to ROS [20]. These findings support the notion that Prx plays an important role in the detoxification of ROS in *S. schenckii*.

It should be noted that, in the present study, three proteins associated with the production of trehalose were differentially expressed; these were the enzymes TreS, Thr, and Tps1. Under conditions of OS, TreS was found upregulated; Thr was exclusively found under these conditions; and Tps1 was shown to be downregulated. These results agree with those reported by Félix-Contreras et al. [13]. Something worth highlighting from this study is that the loss of proteins associated with trehalose synthesis seemed to affect CW integrity and structure, since a decay in β-1,3 glucans has been shown, suggesting an additional regulatory role of the trehalose synthesis proteins over the enzymes related with CW synthesis.

Recent proteomic studies have revealed the presence of numerous putative moonlighthing proteins in the *Candida* CW proteome during OSR [20,21,22]. The moonlighting protein are originally intracellular that perform different functions in addition to their primary housekeeping roles [12]. These proteins lack the typical N-terminal signal peptides for secreted proteins; therefore, a potential mechanism for their nonclassical secretion could be vesicular transport [27]. These proteins are increasingly considered to be important factors in fungal virulence. One hypothesis is that exposed moonlighting proteins in the CW of fungal pathogens have a function in molecular mimicry pathways. These proteins present a high similarity to host proteins, which helps the invading microbes to thwart the host’s immune system [28]

The moonlighting-like proteins identified in this study includes six glycolytic enzymes that were downregulated in *S. schenckii* in the presence of H_2_O_2_. One of these is the Eno, one of the main immunodominant proteins found in the serum of patients with invasive candidiasis [29]. Eno has been found in the CW of several microorganisms (*Bifidobacterium, Staphylococcus aureus*, and *Bacillus anthracis*) and yeasts such as *C. albicans*. The function of Eno in the CW of *Candida* has been associated with its response to OS, because this protein is considered a target of oxidizing agents [30]. Interestingly, it has been shown that in *C. krusei* and *C. glabrata*, Eno is downregulated in the presence of H_2_O_2_ [20], as observed in the present study with *S. schenckii*. Pk is another glycolytic enzyme that has been located on the cell surface of bacteria such as *Lactococcus lactis* [31], and it has also been identified in the serum of a murine model infected with *C. albicans* [32]. The evidence suggests that this enzyme has immunogenic properties, thus, it may interact with the host in response to stress, as reported in *C. albicans* during the OSR [20]. Gapdh has already been identified in the CW of other organisms such as *C. krusei* and *C. parapsilosis* in the presence of the menadione ion (O_2_) and H_2_O_2_ [21]. It has also been identified in the CW of *C. albicans*, and it has been detected in vivo in patients with candidiasis [20]. In *Paracoccidiodes lutzzi*, Gapdh was found at the CW playing a role in fungal adhesion to host tissues and favoring invasion [33]. Fba is an enzyme that presented translational down-regulation in the presence of H_2_O_2_. The Fba enzyme is related to glycolysis. It is also recognized as a protein with dual function and localization, widely reported on the cell surface of many organisms and microorganisms [13,21,34]. Likewise, it has been described in CW studies of *Paracoccidioides* as binding plasminogen, activated to plasmin, representing a potential virulence factor. On the other hand, studies on *Neisseria meningitidis* showed that it could have a function related to adhesion to host cells [33]. It has been reported that it has a decreased expression in other pathogens such as *C. glabrata, C. krusei,* and *C. parapsilosis*, indicating that, like the Gapdh enzyme, this decrease is independent of the phase in which the microorganism is found [20]. In studies by Ramírez Quijas et al. [20] an over-regulation of the Fba1 enzyme was observed in *C. glabrata* under oxidative conditions with menadione (O_2_), demonstrating that Fba1 plays a role in the immune response induced by *Candida* species. Another down-regulated protein involved in the glycolytic pathway and found in the CW of several *Candida* species due to the OSR is Pgk [20,35]. The transcriptional expression of Pgk in *Saccharomyces cerevisiae* was induced through moderate doses of paraquat, in contrast to the excessive doses that gene expression of Pgk [36]. Furthermore, it was shown that recombinant Pgk1 antigens of *C. albicans* reacted to sera from patients with invasive candidiasis infected with various *Candida* species, including *C. albicans, C. tropicalis, C. parapsilosis, C. glabrata, C. lusitaniae, C. krusei,* and *C. guilliermondii* [35]. In another study, it was found that immunization of mice with the recombinant Pgk protein confers protection against *C. auris* [37]. Another glycolytic enzyme that modified its expression was Tpi. Tpi1 plays a role in the interaction between human cells and the CW of *C. albicans* and biofilms formed by these pathogens. Tpi1 was upregulated in ROS and considered as a possible therapeutic target against *C. albicans* [21].

Other metabolic enzymes include Lip1, Adh, and Cs. Lip1 was upregulated, and its participation in the OSR has also been reported in *S. schenckii* in the presence of superoxide ions [13]. Lipases, which are secreted by *C. albicans*, have been described as proteins associated with the CW [38]; they participate in processes such as nutrient acquisition, adhesion to the host cell, and generation of inflammatory mediators [39]. Studies by Toth et al. [40] confirmed the participation of a secreted lipase in the virulence of *C. parapsilosis*. The Cs is associated with the metabolic pathway of tricarboxylic acids. In addition to preserving a dual function and localization, it is described as a moonlighting protein, exposed on the fungal cell surface, playing an important role during adhesion processes to the host cell and evading the immune response by transporting itself from the cytoplasm to the CW through vesicles [13]. Despite the metabolic function of Adh in the cytoplasm, it has been identified as an important factor for the invasion of *Entamoeba histolytica* [41], as a key protein for the construction of biofilms which are necessary for the resistance of *Staphylococcus aureus* [42], and as an allergen and antigen in *C. albicans* [43]. Adh activates THP-1 cells to differentiate into M1 macrophages and acts as an antigenic protein, inducing the host’s innate immune system [44]. Another study indicated that an antibody generated against Adh1 and mAb Ca37, might represent an interesting therapeutic target since it does not bind to the human Adh1 protein [45].

Other moonlighting-like CWPs that modified their expression were Hsp70-5, EF-1β, and EF-1α. Hsp70-5 was upregulated in the presence of H_2_O_2_. The Hsp70 protein family has been largely studied in the yeast *S. cerevisiae* and *C. albicans*, and some of them have been located at the CW [46,47]. These proteins play a key role in the stress response, and are also involved in the translocation of proteins across membranes and protein folding [48,49]. Additionally, they are prominent immunogens during infection [50,51]. EF-1α is highly conserved and plays a central role in protein synthesis within eukaryotic cells [52]. Moreover, EF-1α has a number of other important functions in pathogenesis such as in phagocytosis of *Entamoeba histolytica* [53], and mediating host cell entry by the *Cryptosporidium parvum* parasite, where EF-1α could be a candidate antigen to be used as a vaccine against cryptosporidiosis [54]. In addition, EF-1α plays a role against acute infection by *Toxoplasma gondii*, where the antigen pVAX-EF-1α triggered strong humoral and cellular responses and induced effective protection in mice against acute Toxoplasmosis [55]. Furthermore, EF-1α secreted through extracellular vesicles produced by malaria parasites promotes immune evasion by inhibiting specific T cell responses, and immunization with EF-1α confers antiparasitic protection of long duration and an immune memory [53].

In this study, we determine the gene expression of *HSP70-5, LIP1, ENO*, and *PK* four moonlighting-like protein that were differentially translated. The results showed that transcription was consequent with translation at 15mM H_2_O_2_ condition where *HSP70-5* and *LIP1* were upregulated, while *ENO*, and *PK* were downregulated (Figure 3). However, the effect in transcription was already observed with 5 mM of H_2_O_2_ where all genes tested were upregulated, suggesting the fungi is able to respond at a transcriptional and translational level in order to evade harmful substances such as H_2_O_2_ even at low concentrations. These results are in agreement with susceptibility tests previously reported, where it was observed that H_2_O_2_ began to have an effect at a concentration of 5 mM and greater effects at concentrations of 10, 15, and 20 mM [19]. Proteins expressed after exposure to different concentrations of H_2_O_2_ were studied in *C. albicans, C. glabrata, C. krusei*, and *C. parapsilosis*, and several moonlighting-like CWPs were expressed, including some described in this study such as Eno, Gadph, Pk, Pgk, Fba, and Adh [21]. Additionally, the transcription of ENO was determined, demonstrating that H_2_O_2_ influence transcription and translation of CWPs that participate in the antioxidant mechanisms that protect *Candida* from OS [21]. Eukaryotic cells typically respond to stress conditions by global inhibition of translation [56]. The translation initiation phase is the primary target of regulation and represents a key checkpoint for eukaryotic gene expression. Studies carried out in yeast cells such as *S. cerevisiae* and *C. albicans* have shown a reduction in global protein synthesis in response to OS [57,58]. Our data suggest the possibility that protein synthesis of *S. schenckii* is inhibited in the presence of ROS, but more experiments are required to verify this hypothesis.

According to the results presented above, the identification of CWPs supports the role of these proteins as part of the response to OS induced by H_2_O_2._ These results complement the information obtained in previous studies carried out by our research group [13]. Subsequent studies will focus on elucidating the mechanisms by which CWPs contribute to the virulence of the fungus and its ability to evade the immune response, allowing the pathogen to adhere, infect and remain in human host cells.

## 4. Materials and Methods

### 4.1. Strains and Growth Conditions

*S. schenckii sensu stricto* strain ATCC 58251 was used for this study. To obtain the yeast phase of the fungus, 500 mL Erlenmeyer flasks containing 200 mL of Brain Heart Infusion medium (BHI; Becton Dickinson, Bioxon, Mexico) inoculated with 1 × 10^6^ yeast mL^-1^ and incubated at 37 °C for 72 h (exponential phase) on a rotary shaker at 120 rpm.

### 4.2. Susceptibility Essay with H_2_O_2_

Susceptibility assays with H_2_O_2_ were performed according to the method reported by Ruiz-Baca et al. [17]. Briefly, the cultures were filtered and diluted in fresh BHI medium to obtain an OD_600nm_ of 0.5. They were then divided equally and exposed to different concentrations (0–200 mM) of H_2_O_2_ (Sigma-Aldrich, St. Louis, MO, USA) at 37 °C with stirring (120 rpm) for 60 min. Subsequently, the cultures were centrifuged at 7000× *g* for 10 min, the supernatant was discarded and the pellet was resuspended in 1 mL of deionized water. Exponential dilutions of the cell suspension were performed on 96-well ELISA plates. The sample from each well was seeded in plates with YPD medium and incubated at 28 °C for 4 days.

### 4.3. Extraction of CWPs

To obtain the *S. schenckii* CWPs, the cultures were filtered and distilled water was added to the control cells and 15 mM of H_2_O_2_ to the exposed cells. After 1 h, the cell cultures were centrifuged at 7000× *g* and 4 °C for 10 min and the supernatant was carefully discarded. The pellet was washed three times with lysis buffer [50 mM Tris-HCl, pH 7.5, supplemented with 1 mM phenylmethylsulfonyl fluoride (PMFS)] by centrifuging at 7000× *g* for 10 min in each wash cycle. Yeast cells were disrupted according to the method reported by Ruiz-Baca et al. [17]. The cell homogenate was centrifuged at 7000× *g* at 4 °C for 10 min. The CW pellet was washed with lysis buffer until a clear supernatant was obtained. CWPs were extracted with 2% β-mercaptoethanol and 2% SDS according to the method reported by Félix-Contreras et al. [13]. Protein concentration was determined by DC method (Bio-Rad, Hercules, CA, USA).

### 4.4. Absolute Quantitation by Mass Spectrometry Analysis LC-ESI-IMS- QTof

First, 50 µg of protein per condition was enzymatically digested “in-solution” according to the modified protocol of Zuccoli et al. [59]. A tryptic digested, as an internal standard, of Bovine Serum Albumin (BSA) from Bos taurus (Uniprot accession: P02769) was added to all peptide samples to obtain a final concentration of 25 fmol µL^−1^. Then, peptides were injected using an UPLC ACQUITY M-Class to the mass spectrometer Synapt G2-Si (Waters, Milford, MA, USA) in multiplexed MS/MS (MSE) mode with the objective of calculate the area under the curve (AUC) of the chromatograms in both conditions; and in this way, be able to normalize the injection and introduce the same amount of tryptic peptides in both conditions to the mass spectrometer operated using Data-Independent Acquisition (DIA) and ion mobility spectrometry (IMS) in High-Definition Multiplexed MS/MS (HDMSE) mode. Chromatographic conditions in HDMSE mode were applied according with Félix-Contreras et al. [13], and the conditions for the tune page at the ion source, were applied in according with Landa-Galván et al. [60]. Chromatograms were acquired (low and high energy chromatograms) in positive mode comprising a range of *m*/*z* 50–2000 with a scan time of 500 ms; precursors ions were fragmented using a collision energy ramp through 19–55V. Afterward, the obtained *.raw files were analyzed using DriftScope v2.8 (Waters, Milford, MA, USA) in order to extract the Drif time of each detected peptide in HDMSE as a *.rul file and create a method in Ultra-Definition Multiplexed MS/MS (UDMSE) mode which applies specific collision energy for every peptide detected in this modality (instead of a linear ramp as in the HDMSE mode). The chromatographic and source conditions in UDMSE mode were the same as in HDMSE.

### 4.5. Data Analysis

Generated *.raw files (in UDMSE mode) containing MS and MS/MS spectra were analyzed and absolutely quantified using ProteinLynx Global SERVER (PLGS) v3.0.3 software (Waters), using a target decoy strategy against a *Sporothrix schenckii* ATCC 58251 *fasta database (downloaded from Uniprot 8673 protein sequences, last modified on 26 February 2018) which was concatenated with the same *fasta file in reverse sense. The parameters used for protein identification were as follows: trypsin as cut enzyme and one missed cleavage allowed; carbamidomethyl© as a fixed modification and oxidation (M), amidation (C-terminal), deamidation (Q, N) or phosphorylation (S, T, Y) as variable modifications; peptide and fragment tolerance were set to automatic, minimum fragment ion matches per peptide: 2, minimum fragment ion matches per protein: 5, minimum peptide matches per protein: 1, and false discovery rate ≤ 4%. Synapt G2-Si was calibrated with [Glu1]-fibrinopeptide, [M + 2H]^2+^ = 785.84261 at 1 ppm. All reported proteins in this work presented at least 95% of reliability during the database search (Protein AutoCurate Green). The average intensity of the three most intense peptides per protein (Hi3) was used for the absolute quantitation according with the method described by Silva et al. [61]. All proteins considered differentially expressed display at least a ratio of ±0.585 (expressed as a base 2 logarithm); it means that these proteins had at least ±1.5 absolute fold change. The ratio was calculated based on the absolute quantification (ng on column) of each characterized protein in the “CWP15” sample by absolute quantification (ng on column) of each protein in “CWP0” sample.

### 4.6. Gene Expression Analysis by RT-qPCR

Total RNA was extracted by lysing cell cultures of *S. schenckii* in exponential phase treated with 0, 5, 10, and 15 mM of H_2_O_2_, in liquid nitrogen and using the Trizol method (Thermo Fisher Scientific, Waltham, MA, USA). 1µg of total RNA treated with DNase (Promega, Madison, WI, USA) and quantified using a NanoDrop 2000 (NanoDrop Technologies; Thermo Fisher Scientific, Waltham, MA, USA) was reverse transcribed using the ImProm-II^TM^ Reverse Transcriptase (Thermo Fisher Scientific, Waltham, MA, USA), following the manufacturer´s instructions. Quantitative PCR was performed using the SYBR Green/ROX Maximum qPCR master mix (Thermo Fisher Scientific, Waltham, MA, USA) according to the manufacturer´s instructions, in the Step One real-time system (Thermo Fisher Scientific, Waltham, MA, USA). Primers used for analysis of *HSP70-5, LIP1, ENO*, and *PK* gene expression were designed based on the sequences found in the NCBI [62]. Access numbers and primers for *HSP70-5* (XM_016730833.1), sense 5′-CGCAACGGTCTGGAGAACTA-3′ and antisense 5′-CTGGCTGTACATCTTGCTCG-3′; for *LIP1* (XM_016730213.1), sense 5′-CGAAGAACATTGCCACCTAC-3′ and antisense 5′-GTAGTTGATGTTGACGCCGA-3′; for *ENO* (XM_016730130.1), sense 5′-CCAGCGAGTTCTACAAGGAG-3′ and antisense 5′-CGATCTGGATGTTCTGGGTC-3′; for *PK* (XM_016730089.1), sense 5′- ACCACCGATGACAAGTACGC-3′ and antisense 5′- GTTGACACCCTTCTTGGACG-3′. The housekeeping gene encoding the ribosomal protein L6 (*PL6*) was used as an internal control [63] and the 0 mM H_2_O_2_ condition was used as a calibrator sample. Relative gene expression was calculated using the comparative cycle threshold 2^−^^ΔΔ^^Ct^ method [64].

### 4.7. Statistical Analysis

Statistical analysis for gene expression data was performed using a one-way analysis of variance using ANOVA (one-way ANOVA) and the Bonferroni-Holm post hoc test (Significance: *** *p* < 0.001, ** *p* < 0.01, * *p* < 0.05). The statistical analysis was performed using data from three biological repetitions and three experimental replicates.

## 5. Conclusions

Our results showed that the expression of several CWPs of *S. schenckii* changes during OSR induced by H_2_O_2_. These proteins, together with antioxidant enzymes, could confer protection upon this fungus against ROS generated by the phagocytic cells of the immune system, allowing the fungus to escape from the phagocyte and infect human host cells.

## Figures and Tables

**Table 1:** Expression of CWPs of *S. schenckii* involved in the OSR by H_2_O_2_.

UniProt ID	Protein Name	Max Fold Change	Log2 (Max Fold Change)	Expression	Function
A0A0F2MGW8	Peroxiredoxin (Prx)	6.11	2.61	Upregulated	* Response to stress
A0A0F2LYK0	Superoxide dismutase (Sod) (Cu-Zn)	4.55	2.18	Upregulated	* Response to stress
A0A0F2ME63	GPI-anchored cell wall β-1,3- endoglucanase EglC	3.55	1.81	Upregulated	* Cell wall remodeling
A0A0F2LVB7	β-glucosidase	3.25	1.70	Upregulated	* Cell wall remodeling
A0A0F2MCT9	Covalently-linked cell wall protein	2.69	1.43	Upregulated	* Interaction with the host
A0A0F2MEY8	β-1,3-glucanosyltransferase	2.25	1.17	Upregulated	* Cell wall remodeling
A0A0F2M3E3	Trehalose synthase (TreS)	2.02	1.01	Upregulated	* Trehalose biosynthesis and degradation
A0A0F2M4U6	Heat shock protein 70-5 (Hsp70-5)	2.04	1.32	Upregulated	* Interaction with the host * Response to stress
A0A0F2LUT3	Glycoside hydrolase	2.01	1.01	Upregulated	* Cell wall remodeling
A0A0F2MHR6	GPI anchored cell wall protein	1.93	0.95	Upregulated	* Cell wall remodeling
A0A0F2LWY2	Lipase 1 (Lip1)	1.86	0.90	Upregulated	* Lipid hydrolysis * Interaction with host * Response to stress
A0A0F2M0B7	Glycosidase crf1	1.83	0.87	Upregulated	* Cell wall remodeling
A0A0F2M7G5	Elongation factor 1-beta (EF-1β)	2.84	1.51	Upregulated	* Protein synthesis
A0A0F2MH29	Trehalose-6-phosphate synthase (Tps1)	0.23	−2.09	Downregulated	* Trehalose biosynthesis
A0A0F2MBI8	Citrate synthase (Cs)	0.24	−2.05	Downregulated	* Tricarboxylic acids cycle * Interaction with host cells
A0A0F2M6X5	Glyceraldehyde-3-phosphate dehydrogenase (Gapdh)	0.48	−1.03	Downregulated	* Glycolytic process * Interaction with the host * Response to stress
A0A0F2LYM2	Enolase (Eno)	0.50	−0.99	Downregulated	* Glycolytic process * Interaction with the host * Response to stress
A0A0F2MLK2	Elongation factor 1-alpha (EF-1α)	0.62	−0.67	Downregulated	* Protein synthesis
A0A0F2MJY6	Phosphoglycerate kinase (PgK)	0.63	−0.64	Downregulated	* Glycolytic process * Interaction with the host
A0A0F2LY48	Triosephosphate isomerase (Tpi)	0.64	−0.62	Downregulated	* Glycolytic process * Interaction with the host
A0A0F2LYU3	Fructose-bisphosphate aldolase (Fba)	0.52	−0.93	Downregulated	* Glycolytic process * Interaction with the host
A0A0F2M0W8	Pyruvate kinase (Pk)	NA	Infinity	Exclusive without H_2_O_2_	* Interaction with the host * Glycolytic process * Response to stress
A0A0F2LUL4	β-1,3-glucanosyltransferase	NA	Infinity	Exclusive in H_2_O_2_	* Cell wall remodeling
A0A0F2M6M0	Alcohol dehydrogenase (Adh)	NA	Infinity	Exclusive in H_2_O_2_	* Glycolytic process * Interaction with the host * Biofilm formation
A0A0F2M6S7	Superoxide dismutase (Sod) [Cu-Zn]	NA	Infinity	Exclusive in H_2_O_2_	* Response to stress
A0A0F2M1D9	Trehalase (Trh)	NA	Infinity	Exclusive in H_2_O_2_	* Trehalose biosynthesis
A0A0F2LWR7	Thioredoxin 1 (Thx)	NA	Infinity	Exclusive in H_2_O_2_	* Response to stress
A0A0F2MDI4	CFEM domain-containing protein	NA	Infinity	Exclusive in H_2_O_2_	* Interaction with the host * Biofilm formation

Mass spectra were deconvoluted, compared and quantified using ProteinLynx Global SERVER (PLGS) v3.0.3 software (Waters, Milford, MA, USA) against a reversed *S. schenckii* plus BSA (accession P02769) *.fasta database.

## Data Availability

The datasets generated during and/or analyzed during the current study can be find in the main text and the Supplementary Materials.

## Supplementary Materials

The following are available online at https://www.mdpi.com/article/10.3390/pathogens11020230/s1, Figure S1: Total proteins identified by LC-ESI-IMS- QTof in yeast cells of *S. schenckii* treated or not with H_2_O_2,_ Figure S2: Susceptibility of *S. schenckii* to H_2_O_2_, Data Set S1: Proteomic analysis of *S. schenckii* CW in OSR induced by H_2_O_2_.

## References

[1] Lopez-Romero E., Reyes-Montes M.D.R., Perez-Torres A., Ruiz-Baca E., Villagomez-Castro J.C., Mora-Montes H.M., Flores-Carreón A., Toriello C. (2011). *Sporothrix schenckii* complex and sporotrichosis, an emerging health problem. Future Microbiol..

[2] Chakrabarti A., Bonifaz A., Gutierrez-Galhardo M.C., Mochizuki T., Li S. (2015). Global epidemiology of sporotrichosis. Med. Mycol..

[3] Toriello C., Brunner-Mendoza C., Ruiz-Baca E., Duarte-Escalante E., Pérez-Mejía A., Reyes-Montes M.D.R. (2020). Sporotrichosis in Mexico. Braz. J. Microbiol..

[4] Orofino-Costa R., de Macedo P.M., Rodrigues A.M., Bernardes-Engemann A.R. (2017). Sporotrichosis: An update on epidemiology, etiopathogenesis, laboratory and clinical therapeutics. An. Bras. Dermatol..

[5] Seider K., Heyken A., Lüttich A., Miramon P., Hube B. (2010). Interaction of pathogenic yeasts with phagocytes: Survival, persistence and escape. Curr. Opin. Microbiol..

[6] Erwig L.P., Gow N.A.R. (2016). Interactions of fungal pathogens with phagocytes. Nat. Rev. Microbiol..

[7] Ruiz-Baca E., Perez-Torres A., Romo-Lozano Y., Cervantes-García D., Alba-Fierro C.A., Ventura-Juárez J., Torriello C. (2021). The role of macrophages in the host’s defense against *Sporothrix schenckii*. Pathogens.

[8] Phaniendra A., Jestadi D.B., Periyasamy L. (2015). Free Radicals: Properties, sources, targets, and their implication in various diseases. Indian J. Clin. Biochem..

[9] Halliwell B. (2013). The antioxidant paradox: Less paradoxical now?. Br. J. Clin. Pharmacol..

[10] Hopke A., Brown A.J.P., Hall R.A., Wheeler R.T. (2018). Dynamic fungal cell wall architecture in stress adaptation and immune evasion. Trends Microbiol..

[11] Hernandez-Chavez M.J., Perez-Garcia L.A., Niño-Vega G.A., Mora-Montes H.M. (2017). Fungal strategies to evade the host immune recognition. J. Fungi.

[12] Satala D., Karkowska-Kuleta J., Zelazna A., Rapala-Kozik M., Kozik A. (2020). Moonlighting proteins at the candidal cell surface. Microorganisms.

[13] Felix-Contreras C., Alba-Fierro C.A., Rios-Castro E., Luna-Martinez F., Cuellar-Cruz M., Ruiz-Baca E. (2020). Proteomic analysis of *Sporothrix schenckii* cell wall reveals proteins involved in oxidative stress response induced by menadione. Microb. Pathog..

[14] Andreyev A.Y., Kushnareva Y.E., Starkov A.A. (2005). Mitochondrial metabolism of reactive oxygen species. Biochemistry.

[15] Halliwell B., Gutteridge J.M.C. (1984). Oxygen toxicity, oxygen radicals, transition metals and disease. Biochem. J..

[16] Li P.-F., Dietz R., Von Harsdorf R. (1997). Differential effect of hydrogen peroxide and superoxide anion on apoptosis and proliferation of vascular smooth muscle cells. Circulation.

[17] Ruiz-Baca E., Leyva-Sanchez H., Calderon-Barraza B., Esquivel-Naranjo U., Lopez-Romero E., Lopez-Rodríguez A., Cuéllar-Cruz M. (2019). Identification of proteins in *Sporothrix schenckii sensu stricto* in response to oxidative stress induced by hydrogen peroxide. Rev. Iberoam. Micol..

[18] Cuellar-Cruz M., Briones-Martin-Del-Campo M., Cañas-Villamar I., Montalvo-Arredondo J., Riego-Ruiz L., Castaño I., Penas A.D.L. (2008). High resistance to oxidative stress in the fungal pathogen *Candida glabrata* is mediated by a single catalase, Cta1p, and is controlled by the transcription factors Yap1p, Skn7p, Msn2p, and Msn4p. Eukaryot. Cell.

[19] Roman-Casiano K.M., Martínez-Rocha A.L., Romo-Lozano Y., Lopez-Rodríguez A., Cervantes-García D., Sierra-Campos E., Cuéllar-Cruz M., Ruiz-Baca E. (2021). Enzyme activity and expression of catalases in response to oxidative stress in *Sporothrix schenckii*. Microb. Pathog..

[20] Ramirez-Quijas M.D., Lopez-Romero E., Cuellar-Cruz M. (2015). Proteomic analysis of cell wall in four pathogenic species of *Candida* exposed to oxidative stress. Microb. Pathog..

[21] Serrano-Fujarte I., Lopez-Romero E., Cuellar-Cruz M. (2016). Moonlight-like proteins of the cell wall protect sessile cells of *Candida* from oxidative stress. Microb. Pathog..

[22] Vazquez-Fernandez P., Lopez-Romero E., Cuellar-Cruz M. (2021). A comparative proteomic analysis of *candida* species in response to the oxidizing agent cumene hydroperoxide. Arch. Microbiol..

[23] Mouyna I., Fontaine T., Vai M., Monod M., Fonzi W.A., Diaquin M., Popolo L., Hartland R.P., Latgé J.-P. (2000). Glycosylphosphatidylinositol-anchored glucanosyltransferases play an active role in the biosynthesis of the fungal cell wall. J. Biol. Chem..

[24] Li M., Liu X., Liu Z., Sun Y., Liu M., Wang X., Zhang H., Zheng X., Zhang Z. (2016). Glycoside hydrolase MoGls2 controls asexual/sexual development, cell wall integrity and infectious growth in the rice blast fungus. PLoS ONE.

[25] Perez A., Ramage G., Blanes R., Murgui A., Casanova M., Martínez J.P. (2011). Some biological features of *Candida albicans* mutants for genes coding fungal proteins containing the CFEM domain. FEMS Yeast Res..

[26] Ortega I., Felipe M.S.S., Vasconcelos A.T.R., Bezerra L.M.L., Dantas A.D.S. (2014). Peroxide sensing and signaling in the *Sporothrix schenckii* complex: An *in silico* analysis to uncover putative mechanisms regulating the Hog1 and AP-1 like signaling pathways. Med. Mycol..

[27] Karkowska-Kuleta J., Kulig K., Karnas E., Zuba-Surma E., Woznicka O., Pyza E., Kuleta P., Osyczka A., Rapala-Kozik M., Kozik A. (2020). Characteristics of extracellular vesicles released by the pathogenic yeast-like fungi *Candida glabrata*, *Candida parapsilosis* and *Candida tropicalis*. Cells.

[28] Franco-Serrano L., Cedano J., Perez-Pons J.A., Mozo-Villarias A., Piñol J., Amela I., Querol E. (2018). A hypothesis explaining why so many pathogen virulence proteins are moonlighting proteins. Pathog. Dis..

[29] Lain A., Elguezabal N., Amutio E., de Larrinoa I.F., Moragues M.D., Ponton J. (2008). Use of recombinant antigens for the diagnosis of invasive candidiasis. Clin. Dev. Immunol..

[30] Lu N., Zhang Y., Li H., Gao Z. (2010). Oxidative and nitrative modifications of α-enolase in cardiac proteins from diabetic rats. Free Radic. Biol. Med..

[31] Katakura Y., Sano R., Hashimoto T., Ninomiya K., Shioya S. (2010). Lactic acid bacteria display on the cell surface cytosolic proteins that recognize yeast mannan. Appl. Microbiol. Biotechnol..

[32] Pitarch A., Díez-Orejas R., Molero G., Pardo M., Sánchez M., Gil C., Nombela C. (2001). Analysis of the serologic response to systemic *Candida albicans* infection in a murine model. Proteom. Int. Ed..

[33] Araújo D.S., Lima P.D.S., Baeza L.C., Parente A.F.A., Bailão A.M., Borges C.L., Soares C.M.D.A. (2017). Employing proteomic analysis to compare *Paracoccidioides lutzii* yeast and mycelium cell wall proteins. Biochim. Biophys. Acta (BBA)-Proteins Proteom..

[34] Satala D., Satala G., Karkowska-Kuleta J., Bukowski M., Kluza A., Rapala-Kozik M., Kozik A. (2020). Structural insights into the interactions of candidal enolase with human vitronectin, fibronectin and plasminogen. Int. J. Mol. Sci..

[35] He Z.X., Chen J., Li W., Cheng Y., Zhang H.P., Zhang L.N., Hou T.W. (2015). Serological response and diagnostic value of recombinant *Candida* cell wall protein enolase, phosphoglycerate kinase, and β-glucosidase. Front. Microbiol..

[36] Vassallo N., Galea D.R., Bannister W.H., Balzan R. (2000). Stimulation of yeast 3-phosphoglycerate kinase gene promoter by paraquat. Biochem. Biophys. Res. Commun..

[37] Rosario-Colon J., Eberle K., Adams A., Courville E., Xin H. (2021). *Candida* cell-surface-specific monoclonal antibodies protect mice against *Candida auris* invasive infection. Int. J. Mol. Sci..

[38] Chaffin W.L., López-Ribot J.L., Casanova M., Gozalbo D., Martínez J.P. (1998). Cell wall and secreted proteins of *Candida albicans*: Identification, function, and expression. Microbiol. Mol. Biol. Rev..

[39] Gácser A., Trofa D., Schäfer W., Nosanchuk J.D. (2007). Targeted gene deletion in *Candida parapsilosis* demonstrates the role of secreted lipase in virulence. J. Clin. Investig..

[40] Toth R., Toth A.T.R., Vagvolgyi C., Gacser A. (2017). *Candida parapsilosis* secreted lipase as an important virulence factor. Curr. Protein Pept. Sci..

[41] Ahn C.-S., Kim J.-G., Shin M.H., Lee Y.A., Kong Y. (2018). Comparison of secretome profile of pathogenic and non-pathogenic *Entamoeba histolytica*. Proteomics.

[42] Piras C., Ciccio P.D., Soggiu A., Greco V., Tilocca B., Costanzo N., Ceniti C., Urbani A., Bonizzi L., Ianieri A. (2021). *S. aureus* biofilm protein expression linked to antimicrobial resistance: A proteomic study. Animals.

[43] Swoboda R.K., Bertram G., Hollander H., Greenspan D., Greenspan J.S., Gow N.A., Gooday G.W., Brown A.J. (1993). Glycolytic enzymes of *Candida albicans* are nonubiquitous immunogens during candidiasis. Infect. Immun..

[44] Liu Y., Ou Y., Sun L., Li W., Yang J., Zhang X., Hu Y. (2019). Alcohol dehydrogenase of *Candida albicans* triggers differentiation of THP-1 cells into macrophages. J. Adv. Res..

[45] Antoran A., Aparicio-Fernandez L., Pellon A., Buldain I., Martin-Souto L., Rementeria A., Ghannoum M.A., Fuchs B.B., Mylonakis E., Hernando F.L. (2020). The monoclonal antibody Ca37, developed against *Candida albicans* alcohol dehydrogenase, inhibits the yeast *in vitro* and *in vivo*. Sci. Rep..

[46] Lopez-Ribot J.L., Chaffin W.L. (1996). Members of the Hsp70 family of proteins in the cell wall of *Saccharomyces cerevisiae*. J. Bacteriol..

[47] López-Ribot J.L., Alloush H.M., Masten B.J., Chaffin W.L. (1996). Evidence for presence in the cell wall of *Candida albicans* of a protein related to the hsp70 family. Infect. Immun..

[48] Craig E.A. (2018). Hsp70 at the membrane: Driving protein translocation. BMC Biol..

[49] James P., Pfund C., Craig E.A. (1997). Functional specificity among Hsp70 molecular chaperones. Science.

[50] Kaufmann S.H.E., Schoel B., Morimoto R.I., Tissieres A., Georgopoulos C. (1994). Heat shock proteins as antigens in immunity against infection and self. The Biology of Heat Shock Proteins and Molecular Chaperones.

[51] Maresca B., Kobayashi G.S. (1994). Hsp70 in parasites: As an inducible protective protein and as an antigen. Experientia.

[52] Condeelis J. (1995). Elongation factor 1α, translation and the cytoskeleton. Trends Biochem. Sci..

[53] Demarta-Gatsi C., Rivkin A., Di Bartolo V., Peronet R., Ding S., Commere P.H., Guillonneau F., Bellalou J., Brûlé S., Karam P.A. (2019). Histamine releasing factor and elongation factor 1 alpha secreted via malaria parasites extracellular vesicles promote immune evasion by inhibiting specific T cell responses. Cell. Microbiol..

[54] Matsubayashi M., Teramoto-Kimata I., Uni S., Lillehoj H.S., Matsuda H., Furuya M., Tani H., Sasai K. (2013). Elongation factor-1α is a novel protein associated with host cell invasion and a potential protective antigen of *Cryptosporidium parvum*. J. Biol. Chem..

[55] Wang S., Wang Y., Sun X., Zhang Z., Liu T., Gadahi J.A., Hassan I.A., Xu L., Yan R., Song X. (2015). Protective immunity against acute toxoplasmosis in BALB/c mice induced by a DNA vaccine encoding toxoplasma gondii elongation factor 1-alpha. BMC Infect. Dis..

[56] Proud C.G. (2005). eIF2 and the control of cell physiology. Semin. Cell Dev. Biol..

[57] Sundaram A., Grant C.M. (2014). Oxidant-specific regulation of protein synthesis in *Candida albicans*. Fungal Genet. Biol..

[58] Shenton D., Grant C.M. (2003). Protein S-thiolation targets glycolysis and protein synthesis in response to oxidative stress in the yeast *Saccharomyces cerevisiae*. Biochem. J..

[59] Zuccoli G.S., Martins-De-Souza D., Guest P.C., Rehen S.K., Nascimento J.M. (2017). Combining patient-reprogrammed neural cells and proteomics as a model to study psychiatric disorders. Proteomic Methods Neuropsychiatr. Res..

[60] Landa-Galvan H.V., Rios-Castro E., Romero-Garcia T., Rueda A., Olivares-Reyes J.A. (2020). Metabolic syndrome diminishes insulin-induced Akt activation and causes a redistribution of akt-interacting proteins in cardiomyocytes. PLoS ONE.

[61] Silva J.C., Gorenstein M.V., Li G.-Z., Vissers J.P.C., Geromanos S.J. (2006). Absolute quantification of proteins by LCMSE: A virtue of parallel MS acquisition. Mol. Cell. Proteom..

[62] NCBI Nucleotide Database. https://www.ncbi.nlm.nih.gov.

[63] Trujillo-Esquivel E., Martinez-Alvarez J.A., Clavijo-Giraldo D.M., Hernandez N.V., Flores-Martinez A., Ponce-Noyola P., Mora-Montes H.M. (2017). The *Sporothrix schenckii* gene encoding for the ribosomal protein L6 Has constitutive and stable expression and works as an endogenous control in gene expression analysis. Front. Microbiol..

[64] Schmittgen T.D., Livak K.J. (2008). Analyzing real-time PCR data by the comparative C(T) method. Nat. Protoc..

