# Supplementary material for: Proteomic Analysis of Sporothrix schenckii Exposed to Oxidative Stress Induced by Hydrogen Peroxide
Source: Pathogens. 2022 Feb 10;11(2):230. doi: 10.3390/pathogens11020230 (PMC8880468; doi:10.3390/pathogens11020230)
Supplement: Supplementary file 1 [file pathogens-11-00230-s001.zip › Supplementary Material.pdf]

Figure S1

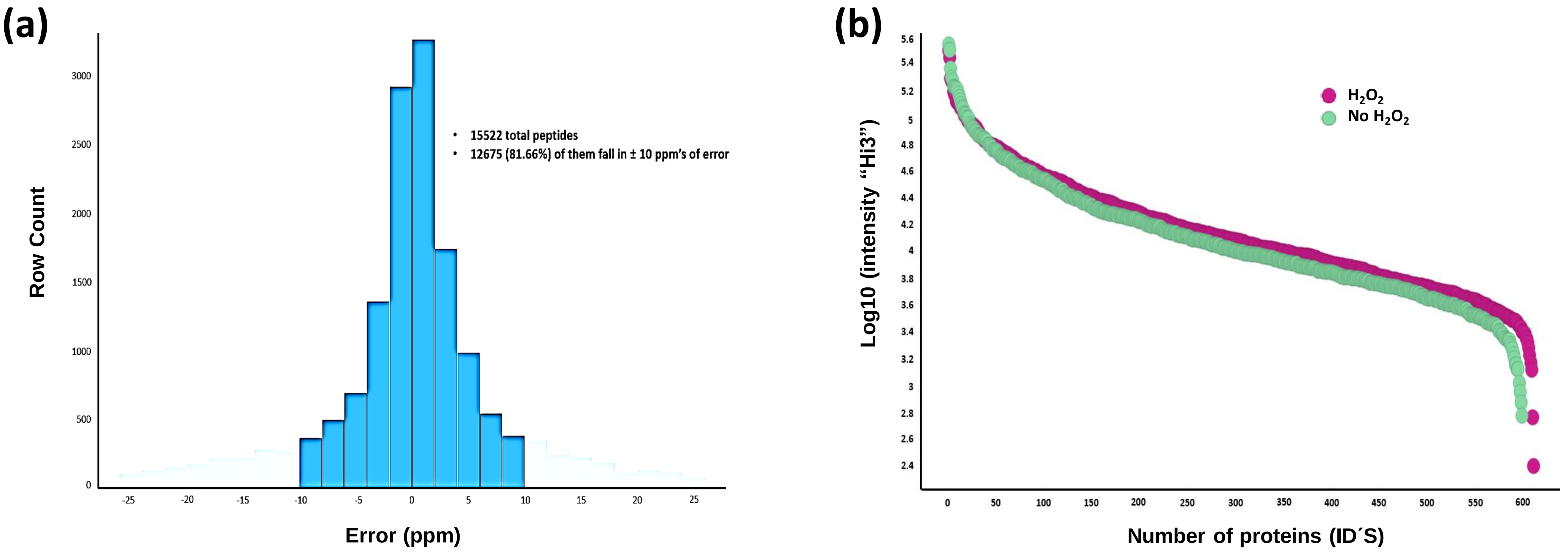

Figure S1. Total proteins identified by LC-ESI-IMS- QTof in yeast cells of *S. schenckii* treated or not with H_20_2. (**a**) The error histogram. (**b**) The dynamic range of proteins in both conditions.

Figure S2

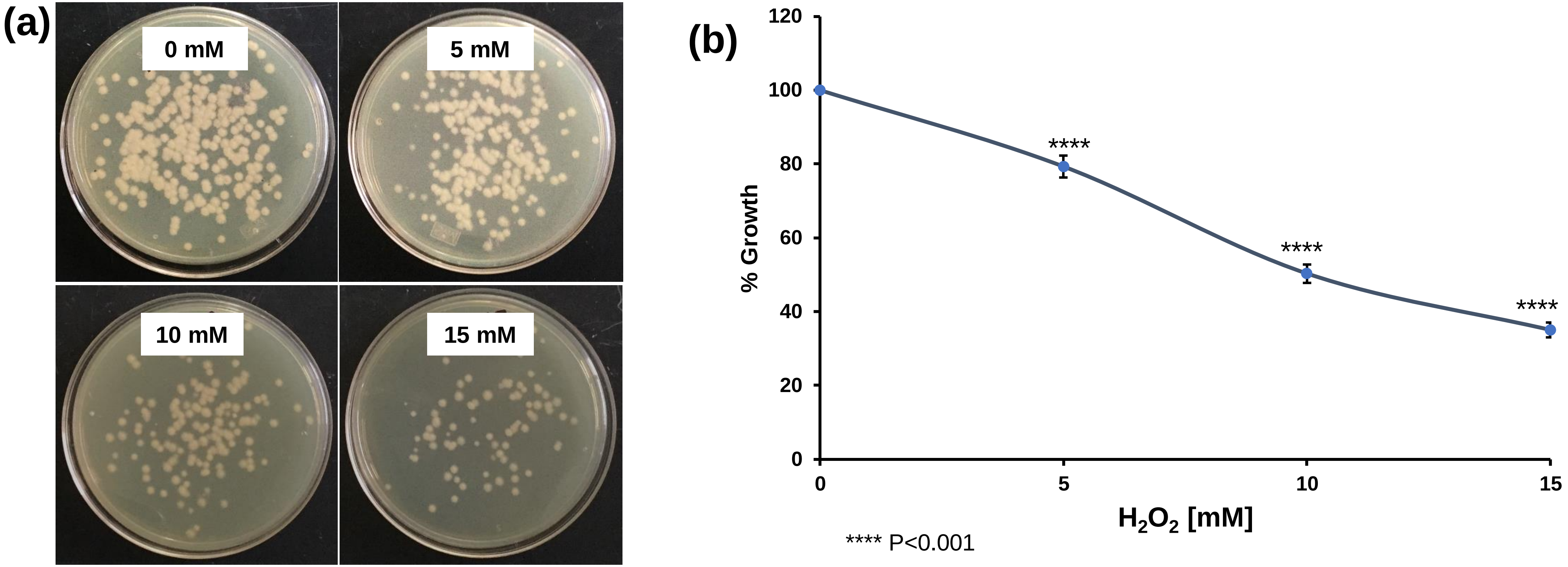

Figure S2. Susceptibility of *S. schenckii* to H_2O_2. Cultures of exponential-phase yeast cell (OD $_{600nm}$ 0.5) were incubated under constant stirring in the presence of 0, 5, 10, and 15 mM of H_2O_2 at 37°C. Samples of these suspensions were diluted to 1 X 10^3 cell mL^{-1}, seeded in plates with YPG medium, and incubated at 37°C. Growth was inspected after 48 h. (a) Plate count, and (**b**) survival %.
